# Living labs for a mobile app-based health program: effectiveness of a 24-week walking intervention for cardiovascular disease risk reduction among female Korean-Chinese migrant workers: a randomized controlled trial

**DOI:** 10.1186/s13690-022-00941-z

**Published:** 2022-08-04

**Authors:** Youlim Kim, Hyeonkyeong Lee, Misook Lee Chung

**Affiliations:** 1 College of Nursing, Kosin University, Busan, South Korea; 2 Mo-Im Kim Nursing Research Institute and Brain Korea 21 FOUR Project, College of Nursing, Yonsei University, Seoul, South Korea; 3 College of Nursing, University of Kentucky, Lexington, KY USA

**Keywords:** Cardiovascular disease, Walking, Exercise, Transients and migrants, Psychological theory

## Abstract

**Background:**

The risk of Cardiovascular disease (CVDs) among adult populations is influenced by environmental factors, and immigrant populations tend to be more vulnerable. This study examined the effectiveness of a 24-week walking program based on social-cognitive determinants through mobile app for CVD risk reduction among female Korean-Chinese middle-aged workers.

**Methods:**

This study used a parallel randomized controlled trial. Participants were recruited by distributing posters and flyers and randomly assigned to either the standard treatment (ST, *n* = 22) or enhanced treatment group (ET, *n* = 28). Participants were provided with a mobile app linked to Fitbit Alta for 24 weeks and instructed to walk at least 30 minutes five times a week and moderate-intensity physical activity. The ET group had additional interventions that enhanced social-cognitive determinants such as self-efficacy, social support. All participants were guided to voluntary physical activity during the 12-week maintenance period. Data were analyzed by the Mann Whitney U-test and a generalized estimating equation.

**Results:**

There were significant between-group differences regarding the number of steps (B = 1.295, *P* < .001) and moderate physical activity time (OR = 6.396, *P* = .030) at week 12. ET group had significant changes in high-density lipoprotein cholesterol (B = 10.522, *P* = .007), low-density lipoprotein cholesterol (B = -16.178, *P* = .024), total cholesterol (B = -20.325, *P* = .039), fasting blood sugar (B = − 8.138, *P* = -.046). In addition, there was a significant reduction of 10-year CVD risk for the ET group over 12 weeks compared to the ST group (B = -0.521, *P*<. 001).

**Conclusions:**

Long-term studies are needed to reduce the risk of cardiovascular disease in large-scale migrant workers and to confirm the direct and insdirect effects of social-cognitive determinants on health outcomes.

**Trial registration:**

The trial was retrospectively registered in WHO ICTRP (KCT0006467) August 19th, 2021. (https://trialsearch.who.int/Trial2.aspx?TrialID=KCT0006467,

## Background

The World Health Organization reported that an estimated 17.9 million people died from cardiovascular disease (CVD) in 2016, representing 31% of all global deaths [1]. The risk of the occurrence of CVDs among various adult populations is heavily influenced by environmental factors [2]. Particularly, immigrant populations tend to have more CVD risk factors, thereby making them more vulnerable to CVD [2]. Particularly, the short-term mortality rate from coronary heart disease is high among Chinese immigrants, which is likely due to inadequate healthcare and difficulty in using medical services [3]. In addition, a systematic review of 16 studies of CVD risk factors reported a higher prevalence of diabetes, hypertension, and higher serum cholesterol among Chinese migrants than those living in mainland China [4].

As a minority ethnic group in China, the Korean-Chinese (KC)—individuals of Chinese nationality—are the largest migrant group (27.8%) in South Korea [5] and have expressed a need for education to prompt a healthy lifestyle and cultural adaptation [6]. Despite the high interest in preventive healthcare, lack of exercise time and motivation, and fatigue pose barriers to physical activity (PA) among KC women [7].

Most CVDs can be prevented by addressing behavioral risk factors such as unhealthy dietand PA [1]. To prevent and improve the risk factors for CVD, including high blood pressure, and hyperlipidemia, health promotion behaviors, such as engagement in PA, should be promoted. The 2019 ACC/AHA Guideline on the Primary Prevention of Cardiovascular Disease in adults recommends 150 minutes/week of moderate-intensity PA (MPA) such as brisk walking [8]. Walking has been demonstrated to reduce cardiovascular risk factors such as blood pressure, body mass index, and total cholesterol (TC) [9, 10].

Previous study [6] has demonstrated the need for interventions to reduce CVD risk and induce a healthy lifestyle in KC migrant women. In particular, there is a need for walking interventions that can be steadily performed without taking much time to monitor one’s health and PA level for cultural specificity of KC middle-aged female workers.

Since social-cognitive approaches (i.e., self-efficacy, social support, outcome expectations, and self-regulation) have been shown to have a positive effect in promoting exercise behavior in PA interventions [11], including walking, it is necessary to apply the social-cognitive theory of walking intervention to prevent CVD in KC women. In addition, mobile health interventions have been reported to be effective in enhancing walking for the reduction of CVD risk [12]. The advantages of mobile health programs are that they can be delivered anywhere, at any time, and for extended periods, thereby facilitating regular communication and behavior maintenance [12]. The interventions, used for various purposes, such as monitoring health status, offering feedback, and providing health information, have been identified as an effective strategy for improving health promotion behavior or preventing disease in general populations [13]. In order to induce health behavior in the context of KC migrant women, it is important to develop a physical activity intervention through participation at all stages, from needs assessment to intervention development and evaluation. Living lab is a practical, open innovation methodology wherein problem-solving methods are co-created through early participation of the target population and that places importance on cooperation among stakeholders [14, 15].

Therefore, it was expected that an mHealth program applying the living lab concept would be an appropriate strategy to provide a walking intervention to KC migrant women. Accordingly, the purpose of this study was to examine the short-term and medium-term effects of a mobile app-based walking intervention on CVD risk outcomes in KC migrant women workers who did not exercise regularly.

## Methods

### Design

**Fig. 1:**
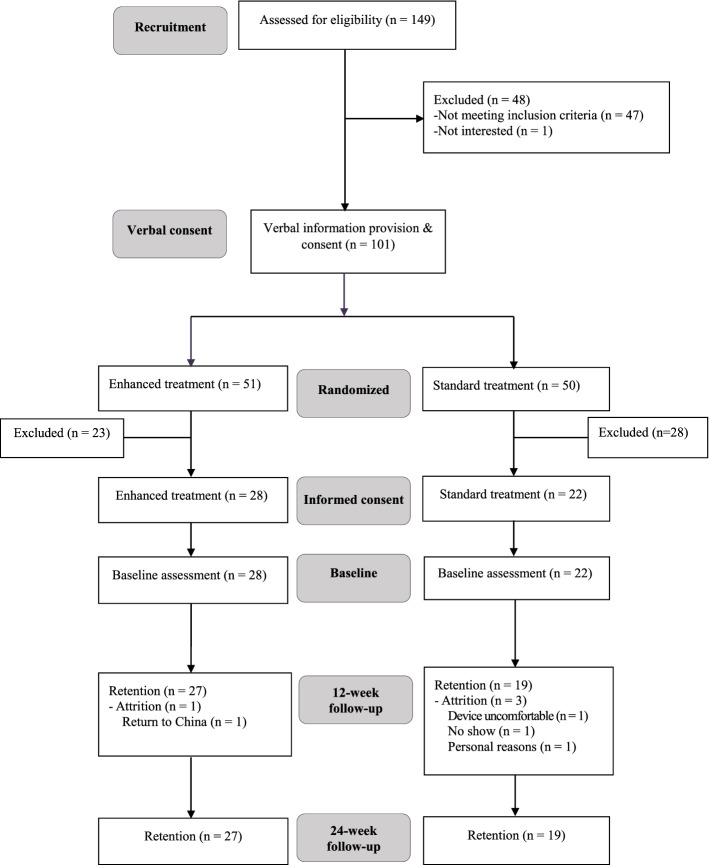
The CONSORT flow diagram for RCT

This study used a parallel randomized controlled trial. Participants were assigned to either the standard treatment (ST) or enhanced treatment (ET) group. The study follows the CONSORT Flow diagram for RCT (Fig. 1) and is registred with cris.nih.go.kr (No. KCT0006467).

### Participants and settings

Eligible participants were KC migrants who (1) were female, (2) were aged between 40 and 65 years, (3) were able to communicate (i.e., speak and write) in Korean, (4) had a full-time job with at least 6 months of working experience, (5) had a smartphone in which mobile apps could be installed, and (6) did not exercise regularly (i.e., 30 minutes a day, more than 3 days a week, for the past 3 months). In addition, we screened for any potential health risks associated with exercise using the Physical Activity Readiness Questionnaire (PAR-Q), which assesses individuals’ minimum readiness for moderate PA programs using seven questions concerning heart disease, chest pain, dizziness, taking blood pressure medications, and bone or joint problems [16]. During the screening test, “For the past 3 months or more, have you been exercising regularly, 3 days a week, 30 minutes a day, or more?” those who responded “Yes” to the question were excluded from the participants.

The G*power 3.1.9.2 program was used to obtain the sample size. As a statistical test, repeated measures, within factors, an effect size of 0.24 was derived from another walking intervention [9] for reducing cardiovascular disease risk among middle-aged Korean-Chinese women workers, a probability of significance of 0.05, a power of 0.8, the number of groups of 2, and the number of measurements 3 times were set. As a result, the number of samples was confirmed to be 30, but considering the dropout rate of 36.4% in the previous study [9], the minimum sample number was set to 50 people.

Eligible participants were recruited for 3 months (April 11 to July 11, 2018). Recruitment was conducted through various organizations in the local community where KC participants were concentrated, such as the church that KC migrants mainly attended, the local public health center and market, and the Korean Foreign Worker Support Center (a local community organization responsible for the employment and welfare of immigrants). In cooperation with community organizations, a poster for recruiting participants was posted on the institution’s bulletin board and website, and flyers were distributed to institutional visitors. In addition, the participants were recruited through those who were trained as health leaders. After face-to-face meetings with leaders of KC women groups, the recruitment flyer was expanded through their Social Networking Service.

### Procedure

This study was approved by the Institutional Review Board of Yonsei University Health System (Y-2018-0057). A single-blinded randomized controlled trial was conducted in Seoul between July 14, 2018 and May 6, 2019. When recruiting participants, trained research assistnats provided overall information on research through face-to-face or telephone interviews. Participants were directly screened for eligibility using the screening test at the recruitment venue or via telephone by trained research assistants. A total of 149 women were screened, of whom 48 were ineligible. After 101 eligible participants verbally consented to participate in this study, they were randomly assigned to either the ET (*n* = 51) or ST (*n* = 50) group. The random allocation was carried out using Random Allocation Software (Ver 1.0, May 2004; available at: https://mahmoodsaghaei.tripod.com/Softwares/randalloc.html) [17]. This software produced as its output a sequence of allocations based on the selected type of blocking and randomly assigned participants to the experimental or control group.

The eligible participants were randomily assignedso that they did not know which group they belonged to, and after 1–2 weeks, they were asked to visit the research facility to complete the written informed consent form and baseline assessment. The principal investigator trained the research assistants regarding the research protocol before data collection. The research assistants then explained to the participants the purpose of the study, highlighting the need for participant engagement, and obtained informed consent. Respondents were informed that participation was voluntary and that they could withdraw at any time. In order to preserve anonymity, participants’ names were not identifiable on any data collection sheets. Before the provision of written informed consent, 51 eligible participants withdrew (ET, *n* = 23; ST, *n* = 28) for various reasons (i.e., time constraints; no show; returned to China; moved to another city; opposition from an employer; uncomfortable using the Fitbit; long-distance). Thus, a total of 50 participants (ET, *n* = 28, ST, *n* = 22) completed the baseline assessment. Of these, 46 completed the 12-week follow-up, and 46 completed the 24-week follow-up.

Trained researchers assistant conducted face-to-face health assessment such as height, weight, lipid profiles, and fasting blood sugar to collect data and investigate general characteristics through structured questionnaires. All data related to health screening (except for demographic characteristics) were measured at baseline, week 12, and week 24. The “Health club” mobile app created by the research team collected the number of walking steps taken the previous day and the duration of moderate-intensity PA of participants through the Fitbit company server. All participants could check these data daily in the “Health club” mobile app, and the research team collected them from the app’s administrator web page.

### Intervention

We developed a living lab for a mobile-based health program focused on improving PA in KC women workers before this study. Living lab principles include multiple methods, user engagement, multi-stakeholder participants, real-life settings, co-creation, and innovation [15]. The final program was a 24-week walking intervention using Fitbit and a mobile app based on social-cognitive theory to prevent CVD risk. Data on the number of walking steps and exercise time of participants wearing the Fitbit were collected through the Fitbit company server, and these data were linked to the ‘Health Club’ app developed by our research team. Details of intervention development are reported elsewhere [6] and summary of the intervention is presented in Table 1.

**Table 1:** Mobile-based health program

	Standard intervention	Enhanced intervention
**12 weeks of adaptation period**	• Providing Fitbit Alta device • Providing mobile app for monitoring walking steps and health information	• Providing Fitbit Alta device • Providing mobile app for monitoring walking steps and health information
		**Social-cognitive intervention**
		• Self-efficacy: setting goal of walking steps, sending a medal image by SNS and, sending short 12 text messages enhancing exerciseefficacy • Social support: sharing information on exercise with participants, supporting each other exercise through SNS • Sense of community: photovoice (i.e., our neighborhood, good place to exercise)
**12 weeks of maintenance period**	• Walking and self monitoring based on mobile app self-walking exercise without intervention from research team	• Walking and self-monitoring based on mobile app without intervention from research team

#### ST Group

All participants were equally guided to walk two steps per second and walk more than half an hour 5 days a week during the 24-week intervention period. Based on the cadence thresholds, which were 100–130 steps/min for absolutely-defined moderate intensity [18], the two steps/second instruction was given so that the participants could easily remember and follow them. They monitored daily walking steps and MPA (minutes of activity over three metabolic task equivalents detected by the wearable device) in a mobile app developed by the research team, connected to the Fitbit Alta [6]. The physical activity data collected by the app via Fitbit Alta was stored on the research team’s computer. A manual explaining the intervention program and a Fitbit user guide were provided, and health check-ups and surveys were conducted three times, including the baseline assessment.

#### ET group

The intervention was preceded by 12 weeks of adaptation; this emphasized social-cognitive determinants (i.e., self-efficacy, social support), and the details of the intervention to the ET group is found on our previous study [6]. In the subsequent 12-week maintenance period, voluntary walking adherence was observed. ET group conducted voluntary walking without the involvement of the researchers in the maintenance period.

Behavioral change techniques, which are known to strengthen positive determinants or mitigate negative determinants affecting behavior change, were used to improve walking adherence [19]. To improve walking-related social support and outcome expectation, the researchers reset walking step goals 3 times every 4 weeks during the 12-week adaptation period, and a medal image was sent via text message to encourage participants whenever they reached the set goals. The ET group, consisting of four teams, selected team leaders in consideration of cultural specificity, and these leaders were guided to encourage social support through Social Networking Service. The ET group received 12 short messages services (less than 60 characters) to encourage walking-related self-efficacy once a week during the 12-week adaptation period (i.e.., “Are you tired often? Exercise promotes blood circulation and helpos to recover from fatigue. Shall we exercise together?”). To improve participants’ sense of community and exercise adherence using community resources, participants were encouraged to upload two photovoice on their SNS account under the themes “good exercise in our neighborhood” and “exercise experience with local people.”In addition, the participants received various awards according to their health-related achievements and level of participation after the intervention finished.

### Measures

#### Adherence to walking

Adherence to walking was assessed in terms of walking steps and MPA time. MPA refers to the “active” Fitbit category (a minimum of 3 metabolic task equivalents or more in at least 10-minute bouts) [20]. The walking step count and MPA time measured on the Fitbit tracker were saved as the previous day’s step count and PA time by linking the data with the app developed by the research team. The research team collected the number of steps and MPA time of the participants every week from the administrator web page linked with the mobile app ‘Health Club’.

An average of 500 steps and more than 4 days of PA recorded through participants’ Fitbit Alta during the seven-day monitoring period were considered a valid number of steps and PA time, respectively [21]. Less than 500 steps were considered an insufficient level of PA, either because of not wearing the device or the device malfunctioning.

#### 10-year risk of CVD

The 10-year risk of CVD was calculated by applying to the Pooled Cohort Equations presented in the 2013 ACC/AHA Guideline on the Assessment of Cardiovascular Risk [22]. To calculate the atherosclerotic CVD (ASCVD) risk score, the variables of sex, race, blood pressure drug use, diabetes status, and smoking status identified in the baseline assessment survey and systolic blood pressure, total cholesterol (TC), high-density lipoprotein cholesterol (HDL-C), and low-density lipoprotein cholesterol (LDL-C) measured in the health check-up were included as predictor variables [22].

#### Lipid profiles and fasting blood sugar

TC, HDL/LDL-C, triglycerides, and blood sugar were measured using the Cholestech LDX Analyzer (Alere, San Diego, CA). Participants were instructed to fast for at least 8 h prior to blood sampling. Experienced nurses collected a small amount (40 μl) of finger capillary blood using a lancet, which was then injected into the examination cartridge. A fasting blood sugar level ≤ 100 mg/dL was considered normal. TC < 200 mg/dL, LDL-C < 130 mg/dL, HDL-C = 40–60 mg/dL, and triglycerides < 150 mg/dL were considered normal [23].

### Statistical analysis

Data were analyzed using SPSS Statistics 25.0 (IBM Corp., Armonk, NY, USA). Descriptive statistics (frequency, percentage, mean, and standard deviation) were used to describe participants’ sociodemographic and health-related characteristics at baseline. As a result of the Shapiro-Wilk test to test the normality of the dependent variable, a non-parametric test was conducted because the assumption of normality distribution was not satisfied. The homogeneity test of variables between the two groups was analyzed with the independent t-test, Mann Whitney U-test, and chi-square test. We divided the average number of steps by 1000 at three-time points (baseline, 12 weeks, and 24 weeks) based on previous studies that combined 1000 steps into one unit [24, 25]. MPA time was converted to binary data based on whether the total weekly duration of PA was more or less than 150 minutes. Since there were missing data at various points in all three assessment stages, the interaction between time and group was confirmed using generalized estimating equations (GEE, link-function: linear, logit) as an intention-to-treat analysis that included missing data. The level of significance was set at 0.05 for all tests.

## Results

### Participants’ sociodemographic and health-related characteristics at baseline

Table 2 shows the participants’ sociodemographic characteristics, walking time, MPA time, and ASCVD risk scores. A homogeneity test was performed to compare sociodemographic characteristics such as age, working time, duration of residence in South Korea, and income for the ET and ST groups. There were significant differences in participants’ age and menopausal status between the ET and ST groups (age: *p* = .014, menopausal status: *p* = .014). There were no significant differences between the groups with regard to PA level, lipid profiles, fasting blood sugar level, and ASCVD risk at baseline.

**Table 2:** Participants’ sociodemographic and health-related characteristics at baseline

	ET (*n* = 28)		ST (*n* = 22)		*x*^*2*^	U	*P*
	n (%)	Mean (SD)	n (%)	Mean (SD)			
**Age**		47.79 (7.01)		53.27 (7.32)		182.0	.014
**Working time (hours a day)**		9.57 (4.40)		9.73 (5.20)		290.0	.729
**Duration of residence (months)**		11.97 (6.27)		13.80 (4.98)		261.0	.358
**Monthly income (USD)**		1486.98 (481.80)		1699.62 (573.05)		280.0	.197
**Education**							
≥ high school	19 (60.7)		14 (63.6)		1.322		.610
**Marital status**							
Married	25 (89.3)		22 (100.0)		2.508		.285
**Job type**							
Service	12 (42.9)		15 (68.2)		4.702		.328
Sales	5 (17.9)		2 (9.1)				
Office job	3 (10.7)		2 (9.1)				
Self-employment	2 (7.1)		2 (9.1)				
Others	6 (21.4)		1 (4.5)				
**Chronic disease**							
Yes	5 (17.9)		8 (36.4)		2.193		.139
**Menopausal**							
Yes	7 (30.4)		13 (68.4)		6.019		.014
**Physical activity**							
Number of steps		10,430.14 (3837.43)		10,422.36 (3164.14)		287.5	.689
MPA time (min/week)		172.32 (129.90)		175.95 (113.52)		292.0	.755
**Blood test**							
Total cholesterol		187.29 (30.73)		192.55 (37.39)		285.0	.653
LDL cholesterol		100.61 (29.16)		93.41 (40.16)		271.0	.470
HDL cholesterol		53.68 (12.59)		58.64 (19.50)		271.5	.475
Triglycerides		148.93 (73.06)		166.23 (63.19)		236.5	.162
Fasting blood sugar		94.26 (10.76)		96.05 (19.99)		270.5	.463
**ASCVD risk**		1.41 (1.23)		2.42 (2.10)		237.0	.164

*ET* Enhanced treatment, *ST* Standard treatment, *SD* Standard deviation, *MPA* Moderate-intensity physical activity, *ASCVD* Atherosclerotic cardiovascular disease

### Comparison of changes in the groups’ PA levels

Table 3 shows the comparison of the groups’ PA levels over time after adjusting for age and menopausal status at baseline. As shown in Table 3, the results of the intention-to-treat approach applying a GEE with linear link function showed significant interaction effects between group and MPA time at 12 weeks. The number of steps in the ET group at 12 weeks significantly increased compared to the number of steps (1000 units) in the ST group (B = 1.295, *P* < .001). The result of the GEE with logit link function analysis according to participants’ MPA time showed a significant interaction effect in the ET group at 12 weeks compared to the ST group at baseline (OR = 6.396, *P* = .030).

**Table 3:** Comparison of changes in physical activity between the two groups

Variables	B / OR	SE	*P*
**Steps/1000**			
Week 12	−.762	.321	.018
Week 24	−.171	.711	.810
ET	−.178	1.192	.882
ET ^a^ Week 12	1.563	.422	<.001
ET ^a^ Week 24	1.295	.950	.173
**MPA time ≥ 150 min (min/week)**			
Week 12	−.672	.696	.511
Week 24	−.372	.809	.646
ET	−.105	.687	.878
ET ^a^ Week 12	6.396	.854	.030
ET ^a^ Week 24	5.965	.931	.055

The model was adjusted for age and menopausal status

^a^indicates interaction of group and time

*SE* Standard error, *ET* Enhanced treatment, *ST* Standard treatment, *MPA* Moderate-intensity physical activity, *OR* Odds ratio

### Comparison of changes in the groups’ health outcomes

Table 4 shows the comparison of health outcomes in the ET and ST groups over time after adjusting for age and menopausal status at baseline.

**Table 4:** Comparison of changes in health outcomes between two groups

Variables	B	SE	*P*
**HDL-C**			
Week 12	−.160	3.203	.960
Week 24	3.263	3.034	.282
ET	−4.747	6.003	.429
ET ^a^ Week 12	10.522	3.914	.007
ET ^a^ Week 24	7.215	4.251	.090
**LDL-C**			
Week 12	5.961	5.132	.245
Week 24	9.684	6.136	.114
ET	2.211	10.529	.834
ET ^a^ Week 12	−16.178	7.161	.024
ET ^a^ Week 24	−4.38	9.854	.657
**Triglycerides**			
Week 12	−23.262	23.253	.317
Week 24	−28.105	19.477	.149
ET	−20.518	23.415	.381
ET ^a^ Week 12	36.212	30.944	.242
ET ^a^ Week 24	17.915	26.320	.495
**TC**			
Week 12	−.262	4.232	.951
Week 24	2.737	6.874	.691
ET	5.933	11.385	.602
ET ^a^ Week 12	−20.325	9.837	.039
ET ^a^ Week 24	−20.911	10.289	.059
**FBS**			
Week 12	4.112	3.295	0.212
Week 24	−2.368	2.579	0.358
ET	−1.117	4.127	0.787
ET ^a^ Week 12	−8.138	4.069	0.046
ET ^a^ Week 24	−1.805	3.485	0.604
**ASCVD risk**			
Week 12	−0.080	0.056	.155
Week 24	−0.343	0.265	.195
ET	−0.200	0.484	.679
ET ^a^ Week 12	−0.521	0.076	<.001
ET ^a^ Week 24	−0.010	0.345	.976

The model was adjusted for age and menopausal status

^a^indicates interaction of group and time

*SE* Standard error, *ASCVD*,Atherosclerotic cardiovascular disease, *ET* Enhanced treatment, *HDL-C* High-density lipoprotein cholesterol, *LDL-C* Low-density lipoprotein cholesterol, *TC* Total cholesterol, *FBS* Fasting blood sugar

In the GEE analysis, the ET group demonstrated significant changes in HDL-C, LDL-C, TC and FBS over 12 weeks compared to the ST group (HDL-C, B = 10.522, *P* = .007; LDL-C, B = − 16.178, *P* = .024; TC, B = − 20.325, *P* = .039, FBS, B = − 8.138, *P* = −.046). However, there were no significant differences in triglyceride change. There was a significant reduction in the primary health outcome—ASCVD risk—for the ET group over 12 weeks compared to the ST group (B = − 0.521, *P* <. 001).

## Discussion

This study first examined the 24-week app-based walking program to strengthen social-cognitive determinants in female KC middle-aged migrant workers to improve their PA adherence and prevent CVD risk. The ET group, which received an enhanced app-based walking intervention and strategies for improving exercise-related self-efficacy and social support, showed a significant reduction in 10-year CVD risk compared to the ST group at week 12, which received only the app-based walking intervention. Our study highlighted that community-based PA interventions that reinforce social-cognitive aspects using wearable devices could effectively prevent CVD risk in a minority population. Similar to previous studies of PA interventions for migrant workers that employed enhanced social-cognitive strategies such as motivation, feedback, role modeling, and social support to improve participants’ exercise-related self-efficacy and foster health behavior changes [26, 27], the present findings support the effectiveness of social-cognitive interventions that enhance self-efficacy, social support, and a sense of community for PA improvement and ultimately CVD risk reduction in KC migrant women.

Previous studies of PA interventions for middle-aged KC women mainly working as housekeepers, restaurant workers, and caregivers have primarily used pedometer-based walking programs [9, 25]. However, a limitation of these studies was that data on PA were collected by self-report methods, such as maintaining a walking diary. In this study, using a wearable device made it possible to improve the accuracy of the collected data and reduce participants’ burden and bias related to self-reported data collection. The wearable device’s close contact with the body made it possible to measure PA level accurately and obtain quantitative data continuously and in real-time [28]. In particular, considering the occupational characteristics of KC middle-aged women who are unable to allot time for exercise after work, Fitbit has the advantage of being able to monitor PA level during daily life activities, occupational activities in the home, and community environments, and housework. Similar to other studies that reported positive health outcomes for Filipino-Americans [29] and African-American women [30] using a mobile app and Fitbit, this trial was meaningful as the mobile app and wearable device were suited to the participants in this study.

In this study, the social-cognitive intervention for the ST group was given only during the adaptation period (weeks 1–12) and participants were encouraged to engage in PA by themselves during the maintenance period (weeks 13–24). As a result, improvements in health outcomes for the ET group were only significant at week 12 compared to the ST group, and no significant effects were observed at week 24 between both groups. Studies that employed the same design to examine PA effectiveness for a Latino ethnic group [31] and African-American women [32] showed similar results. Although the mobile-based social-cognitive intervention had an effect on the increase in the physical activity for 12 weeks and its health outcomes, it can be interpreted. That this effect was not sufficient to sustain physical activity.

In a study that reported modest improvements in PA levels in African-American women in the maintenance period after an active intervention phase, strategies based on a strong behavioral theory that integrated various social-cognitive determinants including self-efficacy, decisional balance, perceived enjoyment of PA, social support from friends and family, and self-regulation were applied [33]. Additionally, incentives were provided to those who completed the monthly surveys even during the six-month maintenance period. It must be noted that the present study did not collect data on participants’ health outcome expectations or provide financial or motivational incentives during the 12-week adaptation period. Studies indicate that health outcome expectations are also related to an individual’s self-efficacy for overcoming the barriers to health behavior from the perspective of social-cognitive theory [34]. Moreover, health outcome expectations motivate practicing healthy behaviors and an incentive for achieving positive health outcomes [35, 36]. In particular, when providing culturally tailored interventions for minority groups, health outcome expectations, as well as health beliefs and values, should be considered [37]. These determinants may influence the interaction between the researcher providing the intervention, the participants receiving it, and the associated health outcomes [38]. Thus, when conducting long-term walking intervention studies on migrants, challenges related to adherence to health behaviors can be expected; however, during the early intervention period, people are more likely to continue to participate in the program, especially if health outcome expectations and financial or motivational incentives are provided [39, 40].

This study has several limitations. For the convenience of recruiting migrant women, which is a hard-to-reach group in the community, KC migrant women who verbally expressed their intention to participate at the recruitment site were selected as participants and randomly assigned. After providing verbal informed consent at the baseline assessment, the women participated in the intervention. However, subsequently, a number of women refused to participate for various reasons in initial phase of the study. The researcher’s physical presence in a face-to-face setting may also be more likely to consent to research participation as a perceived social pressure to conform [41]. Therefore, it is important to form suitable conditions such as a comfortable place when recruiting participants and to provide ample time so that they can consider whether they can afford to participate in the research. A literature review of strategies for sampling, recruitment, and participation in health research on socially vulnerable groups recommends a sufficient period of time for recruiting planned targets, building partnerships with community institutions, strengthening networks, and using tailored individualized approaches for follow-up [42]. Future health intervention research for socially inaccessible groups such as migrants warrants more sufficient recruitment periods and having a community leader in building networks.

Meanwhile, the systematic and meta analyses show that intervention strategies based on multiple social-cognitive determinants have a positive influence on PA and health outcomes [43–45]. However, this study has limitations in generalizing the results in that the improvement level in health outcomes according to the increase in social-cognitive determinants (such as self-efficacy and social support) for increased PA was not confirmed. Therefore, additional studies are required based on the results of this study that investigate the direct and indirect effects of social/social-cognitive theory-based strategies on PA and health outcomes, and how the effect of improving health outcomes as per social-cognitive determinants and physical activity levels occurs sequentially.

## Conclusion

This mobile app-based 24-week walking intervention along with an objective assessment of PA level using Fitbit were effective for increasing PA, reducing cholesterol levels, and subsequently, the 10-year risk of ASCVD of Korean-Chinese migrant women. However, health outcomes for the group that received enhanced treatment to improve social-cognitive determinants (such as exercise self-efficacy and social support) were only significant at week 12 and not at week 24. Future studies should develop strategies aimed at strengthening social-cognitive determinants for sustaining PA with minimal involvemet of the researchers during the maintenance period through the use of mobile technologies. We recommend that longitudinal studies focusing on migrant workers’ perceived social-cognitive determinants, PA level changes, CVD risk factors reduction, and morbidity associated with CVD be conducted.

## Data Availability

The datasets used and/or analysed during the current study are available from the corresponding author on reasonable request.

## Abbreviations

ASCVD: Atherosclerotic cardiovascular disease
CVD: Cardiovascular disease
ET: Enhanced treatment
FBS: Fasting blood sugar
GEE: Generalized estimating eqs.
HDL-C: High-density lipoprotein cholesterol
KC: Korean-Chinese
LDL-C: low-density lipoprotein cholesterol
MPA: Moderate-intensity physical activity
OR: Odds ratio
PA: Physical activity
ST: Standard treatment
TC: Total cholesterol
